# Tobacco control policies in hospitals before and after the implementation of a national smoking ban in Catalonia, Spain

**DOI:** 10.1186/1471-2458-9-160

**Published:** 2009-05-28

**Authors:** Cristina Martínez, Marcela Fu, Jose M Martínez-Sánchez, Montse Ballbè, Montse Puig, Montse García, Esther Carabasa, Esteve Saltó, Esteve Fernández

**Affiliations:** 1 Tobacco Control & Research Unit, Cancer Prevention and Control Department, Institut Català d'Oncologia-IDIBELL, L'Hospitalet de Llobregat, Spain; 2 Catalan Network of Smoke free Hospitals, L'Hospitalet de Llobregat, Spain; 3 Department of Clinical Sciences, Campus of Bellvitge, Universitat de Barcelona, Barcelona, Spain; 4 Alcohol and Addictions Unit, Hospital Clínic i Provincial, Barcelona, Spain; 5 Psychosocial and Mental Health Nursing Department, Universitat de Barcelona, Barcelona, Spain; 6 Public Health Department, Ministry of Health, Generalitat de Catalunya, Barcelona, Spain; 7 Department of Public Health, Universitat de Barcelona, Barcelona, Spain

## Abstract

**Background:**

Diverse projects and guidelines to assist hospitals towards the attainment of comprehensive smoke-free policies have been developed. In 2006, Spain government passed a new smoking ban that reinforce tobacco control policies and banned completely smoking in hospitals. This study assesses the progression of tobacco control policies in the Catalan Network of Smoke-free Hospitals before and after a comprehensive national smoking ban.

**Methods:**

We used the Self-Audit Questionnaire of the European Network for Smoke-free Hospitals to score the compliance of 9 policy standards (global score = 102). We used two cross-sectional surveys to evaluate tobacco control policies before (2005) and after the implementation of a national smoking ban (2007) in 32 hospitals of Catalonia, Spain. We compared the means of the overall score in 2005 and 2007 according to the type of hospital, the number of beds, the prevalence of tobacco consumption, and the number of years as a smoke-free hospital.

**Results:**

The mean of the implementation score of tobacco control policies was 52.4 (95% CI: 45.4–59.5) in 2005 and 71.6 (95% CI: 67.0–76.2) in 2007 with an increase of 36.7% (p < 0.01). The hospitals with greater improvement were general hospitals (48% increase; p < 0.01), hospitals with > 300 beds (41.1% increase; p < 0.01), hospitals with employees' tobacco consumption prevalence 35–39% (72.2% increase; p < 0.05) and hospitals that had recently implemented smoke-free policies (74.2% increase; p < 0.01).

**Conclusion:**

The national smoking ban appears to increase tobacco control activities in hospitals combined with other non-bylaw initiatives such as the Smoke-free Hospital Network.

## Background

After the approval of the World Health Organization (WHO) Framework Convention on Tobacco Control [1], many countries have introduced smoke-free policies to protect non-smokers from the hazards of secondhand smoke (SHS) [2]. Hospitals should be an example in terms of controlling tobacco consumption and championing compliance with the law [3,4]. Furthermore, health care services should take the lead in implementing smoke-free policies which promote adequate environments for patients, visitors and employees. Current evidence suggests that a comprehensive tobacco policy in hospital settings should include enforcement of indoor smoke-free policies, reducing tobacco consumption among health professionals, encouraging abstinence for patients [5,6], and contributing in health promotion to denormalise tobacco consumption [7].

In 1993, US hospitals became smoke-free in accordance with the Joint Commission on Accreditation of Health Care Organizations [8]. In Europe, the European Network of Smoke-free Hospitals (ENSH) has developed an European Code that sets guidelines for the establishment of smoke-free policies in hospitals since 2000 [9]. Furthermore, the ENSH has developed standards and supportive instruments to assist hospitals' efforts towards the attainment of a comprehensive smoke-free policy [5,10].

The Catalan Network of Smoke-free Hospitals has used the ENSH model to promote smoke-free hospitals in Catalonia, Spain [5]. The national government of Spain passed a new tobacco control law that came into force the first of January 2006 [11]. Smoking was banned in all enclosed public places and workplaces, including health care facilities [12]. Smoking in care centres was already prohibited under both national and regional previous legislation in Catalonia [13], although smoking rooms and smoking areas within the hospitals' cafeterias were allowed. The new law, however, bans completely smoking in all health care facilities without exceptions. After the law, SHS exposure has decreased in Catalan hospitals [14].

This study assesses the progression of tobacco control policies in the Catalan Network of Smoke-free Hospitals before and after a national smoking ban in hospitals that had implemented the ENSH Code and Standards, and hence may contribute to further evaluate the impact of the law.

## Methods

We conducted two independent cross-sectional surveys to monitor tobacco control policies in hospitals members of the Catalan Network of Smoke-free Hospitals at consolidation stage. We defined as consolidation stage those hospitals with two or more years of enrollment after the official launching of the project [5] in 2005. From the 43 members of the Network in 2005, 32 (74.4%) satisfied this criterion, and were included in the study. The baseline survey was run in April 2005, six months before implementing the law, and the second one a year and four months after its implementation in April 2007.

**Figure 1:**
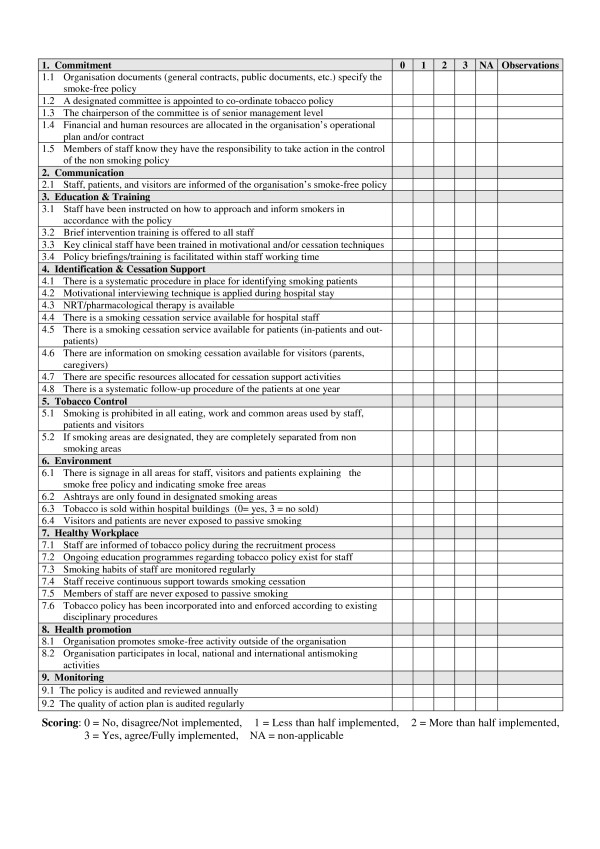
**European Self-Audit Questionnaire (SAQ) for monitoring policy standards at hospitals**.

The degree of implementation of the Smoke-free Hospitals Project was analysed by means of the Self-Audit Questionnaire (SAQ) of the European Network for Smoke-free Hospitals. The SAQ enables hospitals to monitor and review their own progress towards the achievement of a written smoke-free policy that ensures the attainment of a totally smoke-free environment. The SAQ is also a tool to acknowledge and reward continuous improvement by facilitating hospitals to categorize their progress. This instrument was developed to analyse the extent to which tobacco control measures are complied within hospitals [5]. The questionnaire includes 9 standards (see Figure 1) with different number of items: commitment (5 items), communication (1 item), education and training (4 items), identification and cessation support (8 items), tobacco control (2 items), environment (4 items), healthy workplace (6 items), health promotion (2 items), and follow-up (2 items). Each item is scored as follows: 0 = not implemented, 1 = less than half the aspects are implemented, 2 = more than half are implemented, 3 = fully implemented, NA = not applicable. The maximum score of the Self Audit Questionnaire is 102 points, as the sum of its 9 standards. The SAQ was developed by an experts' working group from the ENSH and piloted in smoke-free hospitals in Ireland, France, Finland, and Italy. No formal assessment of its psychometric properties has been done to date, but its feasibility has been tested [15]. The questionnaire was sent by e-mail to tobacco control coordinators in each hospital in April 2005 and April 2007 to be completed and returned to the Network coordinating centre. We gave participating hospitals four weeks to complete the questionnaire by group consensus and submit the results. The response rate was 100% both in 2005 and 2007.

The degree of implementation of the Smoke-free Hospitals Project was analysed by means of the score obtained in the SAQ. For the sake of simplicity, we standardized to 100. We computed the mean and 95% confidence interval (CI) of the overall score and the 9 policies standards included in the SAQ in 2005 and 2007, according to the type of hospital (general, reference and high technology), the number of beds (≤ 300 or > 300), the staff hired in hospitals (≤ 700 or > 700), the prevalence of tobacco consumption (< 30, 30–34, 35–39 and ≥ 40%), surveyed from 2003 to 2005, and the number of years as smoke-free hospital (≤ 4 or > 4). We calculated the percentage of change for the global score and for each policy standard. We used Wilcoxon signed-rank non-parametric test to compare the SAQ scores of hospitals before and after the implementation of the smoking ban.

## Results

We included the 32 hospitals at the consolidation stage of the Smoke-free Hospital project that had completed the SAQ in 2005 and in 2007. Thirteen were general hospitals, 12 reference hospitals, and 7 high technology hospitals. Fifteen hospitals were members of the Network for ≤ 4 years and 17 for > 4 years in 2007.

### Total score of the Self-Audit Questionnaire according to hospital's characteristics before and after the smoking ban

The overall mean of implementation score of tobacco control policies was 52.4 (95% CI: 45.4–59.5) in 2005 and 71.6 (95% CI: 67.0–76.2) in 2007, with an increase from the baseline results obtained in 2005 of 36.7% (p < 0.01) (Table 1).

**Table 1:** Self-Audit score according to hospitals' characteristics before (2005) and after (2007) the implementation of the Spanish tobacco law.

	**2005**		**2007**			
	**Mean**	**95% CI**	**Mean**	**95% CI**	p*	**% Increase**
						
**Overall score **(n = 32)	52.4	(45.4–59.5)	71.6	(67.0–76.2)	< 0.01	36.7
						
**Level of health care**						
General hospital (n = 13)	46.0	(33.3–58.6)	68.1	(59.1–77.3)	< 0.01	48.0
Reference hospital (n = 12)	54.7	(40.7–68.5)	74.2	(68.0–80.4)	< 0.05	35.6
High technology hospital (n = 7)	60.6	(52.2–68.9)	73.5	(61.3–85.8)	< 0.05	21.4
**Beds**						
≤ 300 (n = 16)	52.4	(41.6–63.2)	69.1	(61.4–76.8)	< 0.01	16.7
> 300 (n = 16)	52.5	(42.1–63.0)	74.1	(68.4–79.8)	< 0.01	41.1
**Staff**						
≤ 700 (n = 14)	47.4	(33.7–61.1)	71.7	(62.7–80.8)	< 0.01	51.3
> 700 (n = 18)	56.4	(48.7–64.0)	71.5	(66.3–76.7)	< 0.01	26.7
**Tobacco prevalence**						
< 30% (n = 6)	43.4	(22.9–63.9)	71.7	(62.6–80.7)	< 0.05	65.2
30–34% (n = 16)	63.4	(56.9–70.0)	74.6	(67.0–82.2)	< 0.05	17.6
35–39% (n = 7)	35.8	(17.1–54.6)	61.7	(53.0–70.3)	< 0.05	72.2
≥ 40% (n = 3)	50.7	(-2.8–104)	78.8	(60.8–96.7)	NS	55.4
**Years of adscription (in 2007)**						
≤ 4 years (n = 15)	40.4	(29.0–51.9)	70.4	(62.3–78.4)	< 0.01	74.2
> 4 years (n = 17)	63.0	(57.4–68.7)	72.7	(66.7–78.5)	< 0.05	15.3

CI: confidence interval

NS: no statistically significant p > 0.05

* p-value for Wilcoxon's signed rank test

We observed the highest scores in 2007 in hospitals with smoking prevalence over 40%, with a mean of 78.8 (95% CI: 60.8–96.7), and in reference hospitals with a mean of 74.2 (95% CI: 68.0–80.4). Hospitals with a smoking prevalence between 35–39%, and general and small (≤ 300 beds) hospitals attained the lowest scores (Table 1). By years of enrolment, the mean score obtained in 2005 was 22.6 points higher in those hospitals with > 4 years in the project than in hospitals with ≤ 4 years. However, the difference narrowed to 2.3 points in 2007 between these two groups of hospitals.

By level of health care, the hospitals that improved the most after the application of the national ban were the general hospitals (increase of 48%; p < 0.01), and those that increased less have been the high technology hospitals (increase of 21.4%; p < 0.05) (Table 1).

By number of beds and number of staff hired in hospitals, the increase has been higher in those with > 300 beds (increase of 41.1%; p < 0.01) and with ≤ 700 workers (increase of 51.3%; p < 0.01). Regarding smoking prevalence, hospitals with prevalence rate between 35–39% (increase of 72.2%; p < 0.05) and those with < 30% (increase of 65.2%; p < 0.05) were the ones with higher score increase after the application of the law. By years of implementation of the smoke-free hospital, those with ≤ 4 years of implementation growth the most (increase of 74.2%; p < 0.01).

### Score and increase by policy standards of the Self-Audit Questionnaire before and after the ban

We observed that the scores in all the standards improved after the application of the national smoking ban. The standards with the highest increase were "healthy workplace" (increase of 78.3%; p < 0.01) and "health promotion" (increase of 57.1%; p < 0.05). Moreover, we enclosed standards that almost have achieved their maximum development at Catalan Network of Smoke-free Hospitals such "tobacco control" and "environment" (Table 2).

**Table 2:** Scores according to standards of the SAQ before (2005) and after (2007) the implementation of the Spanish tobacco law.

		**2005**		**2007**			
**Standard**	**Maximum Score available**	**Mean**	**95%CI**	**Mean**	**95%CI**	p*	**% Increase**
**Commitment**	15	9.1	(8.1–10.0)	11.1	(10.1–12.0)	< 0.01	22.5
**Communication**	3	2.3	(1.9–2.7)	2.7	(2.6–2.9)	< 0.01	18.4
**Education and training**	12	4.8	(3.6–6.0)	7.1	(6.1–7.0)	< 0.01	47.6
**Identification and cessation support**	24	8.7	(8.7–12.8)	12.8	(10.5–15.7)	< 0.01	47.1
**Tobacco control**	6	4.7	(4.1–5.5)	5.7	(5.4–6.0)	< 0.05	21.2
**Environment**	12	10.1	(9.1–11.2)	11.8	(11.7–12.0)	< 0.01	16.8
**Healthy workplace**	18	7.4	(5.6–9.2)	13.2	(12.0–14.5)	< 0.01	78.3
**Health promotion**	6	2.1	(1.3–2.9)	3.3	(2.5–4.2)	< 0.05	57.1
**Follow-up**	6	3.7	(2.8–4.6)	5.1	(4.6–5.8)	< 0.05	37.8

CI: confidence interval

* p-value for Wilcoxon's signed rank test

Comparing the results by level of hospitals, we observed a particular situation after the appliance of the tobacco control law, in the results obtained in "education and training" and "identification and cessation support". Whereas hospitals with reference level taught more of their staff in tobacco intervention, with a mean score of 7.5 (95% CI: 6.3–8.7), high technology hospitals offered more cessation program, with a mean score of 17.6 (95% CI: 11.1–23.2; p < 0.05). Finally, hospitals with ≥ 4 years in the network are the ones that apply more cessation programs (mean score = 14.6; 95% CI: 11.5–17.6), and hospitals with < 4 years educated and trained more their staff members (mean score = 7.4; 95% CI: 7.2–14.5) (p < 0.01).

## Discussion

This study indicates how tobacco control policies, as measured by the scores of SAQ, have increased in hospitals after the implementation of a national tobacco control law. The hospitals that have increased the most were the general hospitals, those with > 300 beds, with staff ≤ 700, with tobacco consumption prevalence 35–39%, and with ≤ 4 years of participation. In terms of growth we observed that the highest raises have been produced in those hospitals with an initial worst situation. This could be partly explained by regression towards the mean [16]. However, the increase in SAQ scores was generalized in all hospitals except in four of them (those with the highest scores pre-ban). Hospitals with a shorter enrollment in the Smoke-free Network have achieved similar scores than hospitals with more years in the Network after the enforcing of the ban.

Spain applied like other European countries (Norway, Ireland, Italy, Malta and Sweden) a national law that bans smoking in public places including hospitals. Comparing our results with a multi-country study run by ENSH, Catalonia has achieved a high implementation of the project only overcome by Ireland [17]. Although national and regional partial regulations were previously in force in our country, it is clear that the new comprehensive law has reinforced the accomplishment.

Hospitals members of the Catalan Network have increased their monitoring activities to measure progress toward a smoke-free policy after the implementation of the law. This fact suggests that hospitals identified their weakness to update and increase their quality in the search of the "gold standard".

Since 2004 the Catalan Network of Smoke-free Hospitals monitors the progression of tobacco control policies by means of the SAQ. The results have shown the annual growth of the ENSH standards according this evaluation tool. At the beginning of its use the mean SAQ score was 47.5 (year 2004) and three years later was 71.6. The utmost increase in tobacco control policies was achieved from 2005 to 2006 with a 25.1% increase in the score. This increase is twofold comparing to the preceding year (10.3%) and the observed in the subsequent year (9.2%). This pattern indicates that the new law has an independent effect besides the expected annual increase already observed.

In addition to the increase observed in SAQ scores, hospitals are still suitable to broaden their policies. Some areas that have achieved only 50% of their maximum score possible could be enhanced (i.e., "education and training", "identification and cessation support" and "healthy workplace"). So we should increase and intensify the hospitals' measures addressed to inform and ask for the commitment of the tobacco policies to new staff members, monitor their tobacco consumption, and provide cessation programs inside the institutions. The growth in those areas could be a solution to work out with the lack of support and fulfillment of health professionals in the implementation of smoke-free polices at hospitals showed in other studies [18,19]. Although smoking inside the hospitals is forbidden, there are still areas where SHS is detectable [14]. Policy infringements are common in hospitals and require reinforcement, including measures to control tobacco consumption and to reduce the visibility of health professionals smoking in their white suits [20].

Smoking by patients is still common and craving occurres frequently [21,22]. Therefore smoking care practices, such as identification of smoking status, counseling, and provision of cessation therapy, are necessary [6]. Even in the context of smoke-free hospitals site, the majority of patients who are smokers receive inadequate smoking care [23]. From our study, training and education in tobacco cessation and intervention programs are still areas to enhance. Although previous instructive tobacco cessation initiatives in our context have shown that teaching increases professionals' knowledge of psychological skills and pharmacological resources, no changes have been observed in professionals' attitude in providing help to quit [24]. The lack of systematic protocols to attend smokers at hospitals could be a barrier to apply the knowledge of professionals. Hospital policies should include intervention protocols for all units and services, where all the professionals had the responsibility to tackle the issue as a front line issue in their everyday practice. Without clear and easy protocols regarding cessation there is limited support to integrate cessation into clinical practice [25]. Smoke-free policies should be viewed as a part of large comprehensive strategies, the implementation of which is arguably the most important action of heath prevention, promotion, and recover from illness. The constant strengthening of the smoke-free hospital policy and its active promotion seems a central determinant of successful policies.

Among the potential limitations of the study we should note that the questionnaire has been filled in by the Project's Coordinators, after a consensus meeting with others key persons involved in tobacco control in the hospitals. Therefore, some bias due to self-complacency can not be ruled out. The Catalan Network compares this data with other more objective results, such as the tobacco consumption surveys, airborne nicotine measurements, and observational surveys of tobacco consumption signs. Up to now, the SAQ has not been formally validated against these objective measures, but the observed agreement between them is high.

We should also mention some strengths of this study. We have annually assessed the tobacco control policies at hospitals using the SAQ since 2004, which permits to evaluate both the individual progression of the hospitals and the progression of the Network. In addition, this tool is used by more than 1180 European Hospitals and should allow contrasting our results with other national or regional Networks.

## Conclusion

This research has important public health and policy implications for tobacco control in hospitals. First, we have seen that national smoking bans are effective in combination with other initiatives such as the Catalan Network of Smoke-free Hospitals. Second, the yearly assessment of tobacco control policies by the SAQ helps to identify the strengths and weaknesses in each hospital, so best strategies towards a smoke-free policy can be developed. And third, hospitals should incorporate effective smoking cessation interventions as part of a standard practice. Consequently, tobacco regulations and bans should be accompanied by organizations and resources to guarantee the implementation of policies.

## Competing interests

The authors declare that they have no competing interests.

## Authors' contributions

CM and EF conceived and designed the study. CM supervised the study and data collection, interpreted the data, and wrote the first draft of the manuscript. JMM and MF were responsible for the analysis and interpretation of data. MB, MP, and EC were involved in data collection and with EF, ES and MG revised the manuscript for intellectual content. All authors read and approved the final manuscript.

## Pre-publication history

The pre-publication history for this paper can be accessed here:



## References

[B1] Shibuya K, Ciecierski C, Guindon E, Bettcher DW, Evans DB, Murray CJ, WHO (2003). Framework Convention on Tobacco Control: Development of an evidence based global public health treaty. BMJ.

[B2] Joossen L, Raw M (2007). Progress in tobacco control in 30 European countries 2005 to 2007. 4th European Conference Tobacco or Health 2007: 11–13 October 2007; Basel, Switzerland.

[B3] McKee M, Gilmore A, Novotny TE (2003). Smoke-free hospitals and the role of smoking cessation services. BMJ.

[B4] Neubeck L (2006). Smoke-free hospitals and the role of smoking cessation services. Br J Nurs.

[B5] Garcia M, Mendez E, Martinez C, Peris M, Fernandez E (2006). Implementing and complying with the smoke-free hospitals project in Catalonia, Spain. Eur J Cancer Prev.

[B6] Rigotti NA, Arnsten JH, McKool KM, Wood-Reid KM, Pasternak RC, Singer DE (2000). Smoking by patients in a smoke-free hospital: Prevalence, predictors, and implications. Prev Med.

[B7] Kunyk D, Els C, Predy G, Haase M (2007). Development and introduction of a comprehensive tobacco control policy in a Canadian regional health authority. Prev Chronic Dis.

[B8] UC Joint Comission on Accreditation of Healthcare Organizations (1992). Acreditation manual for hospitals: Joint Comission on Accreditation of Healthcare Organizations, editor.

[B9] ENSH (1999). European Smoke-free Hospital Network Newletter. http://ensh.aphp.fr/index.php?langue=2&language=2.

[B10] Nardini S, Pacifici R, Mortali C, Zuccaro PG (2003). A survey on policies of smoking control in Italian hospitals. Monaldi Arch Chest Dis.

[B11] LEY 28/2005 De 26 De Diciembre, de Medidas Sanitarias frente al tabaquismo y reguladora de la venta, el suministro, el consumo y la publicidad de los productos del tabaco. [Health measures of the selling, use, consumption and publicity of tobacco products] BOE Núm 309 De 27 De Diciembre.

[B12] Fernandez E (2006). Spain: Going smoke free. Tob Control.

[B13] DOGC Catalan Law 10/1991, of 10 may modifying Law 20/1985 de Prevenció i provisió de substances dependets[Prevention and Healthcare Provision for Substances which may Create Dependency]. DOGC 1445 22-05-1991.

[B14] Fernández E, Fu M, Martínez C, Martínez-Sánchez JM, López MJ, Martín-Pujol A, Centrich F, Muñoz G, Nebot M, Saltó E (2008). Secondhand smoke in hospitals before and after a ban on smoking in Catalonia (Spain). Prev Med.

[B15] Ouranou A (2003). Self-audit process and results from preliminar experiences of the ENSH members. European Network Smoke free Hospitals Newsletter.

[B16] Bland JM, Altman DG (1994). Regression towards the mean. BMJ.

[B17] Dauzenberg B (2007). Evaluating compliance and monitoring the progress of 10 European countries using the ENSH self administrated questionnaire in 2006. European Network Smoke-free Hospitals Newsletter.

[B18] Longo DR, Feldman MM, Kruse RL, Brownson RC, Petroski GF, Hewett JE (1998). Implementing smoking bans in American hospitals: Results of a national survey. Tob Control.

[B19] Martinez C, Garcia M, Mendez E, Peris M, Fernandez E (2008). Barriers and challenges for tobacco control in a smoke-free hospital. Cancer Nurs.

[B20] Ratschen E, Britton J, McNeill A (2008). Smoke-free hospitals – the English experience: Results from a survey, interviews, and site visits. BMC Health Serv Res.

[B21] Sabido M, Sunyer J, Masuet C, Masip J (2006). Hospitalized smokers: Compliance with a nonsmoking policy and its predictors. Prev Med.

[B22] Nieto MA, Abdel Kader L, Rosado M, Carriazo A, Arias L (2003). Tabaquismo en pacientes hospitalizados [Tobacco consumption in inpatients]. Anales de Medicina.

[B23] Freund M, Campbell E, Paul C, Sakrouge R, Wiggers J (2005). Smoking care provision in smoke-free hospitals in Australia. Prev Med.

[B24] Ballbe M, Mondon S, Nieva G, Walther M, Salto E, Gual A (2008). Evaluación de un programa de formación de profesionales sanitarios sobre abordaje del tabaquismo en pacientes hospitalizados [Evaluation of a training programme for health professionals on smoking cessation in hospitalized]. Adicciones.

[B25] Schultz AS, Bottorff JL, Johnson JL (2006). An ethnographic study of tobacco control in hospital settings. Tob Control.

